# Assessment of facial analysis measurements by golden proportion^☆^^☆☆^

**DOI:** 10.1016/j.bjorl.2018.07.009

**Published:** 2018-08-18

**Authors:** Kerem Sami Kaya, Bilge Türk, Mahmut Cankaya, Nurullah Seyhun, Berna Uslu Coşkun

**Affiliations:** a Şişli Hamidiye Etfal Training and Research Hospital, Otolaryngology Department, Istanbul, Turkey; b Dr. Burhan Nalbantoğlu State Hospital, Lefkoşa, Cyprus

**Keywords:** Facial analysis, Golden proportion, Facial aesthetic, Análise facial, Proporção áurea, Estética facial

## Abstract

**Introduction:**

The face is the most important factor affecting the physical appearance of a person. In facial aesthetics, there is a specific mathematical proportion, which is called golden proportion, used to measure and analyse facial aesthetic qualities in population.

**Objectives:**

The aim of this study was to measure the facial soft tissue proportions which would help to constitute a standard for facial beauty and diagnose facial differences and anomalies and to compare these proportions to the golden proportion.

**Methods:**

One hundred and thirty-three (133) Turkish patients 18–40 years of age (61 females, 72 males) were involved in the study. Analysis of the photographs was performed by the same physician, and a software programme was used (NIH Image, version 1.62). Facial proportions were measured and differences from the golden proportions were recorded and grouped as normal (1.6–1.699), short (<1.6) and long (>1.699).

**Results:**

According to the facial analysis results, the trichion–gnathion/right zygoma–left zygoma was assessed: 33.1% of the patients were in normal facial morphology, 36.8% were in long facial morphology and 30.1% were in short facial morphology, according to this proportion. The trichion–gnathion/right zygoma–left zygoma proportion was significantly higher in males than females (*p* < 0.001). Statistically significant difference was noted in gender groups, according to the trichion–gnathion/right zygoma–left zygoma and the right lateral canthus–left lateral canthus/right cheilion–left cheilion proportions (*p* = 0.001, *p* = 0.028).

**Conclusion:**

Facial proportion assessments in relation to the golden proportion showed that a statistically significant difference was observed between gender groups. Long facial morphology was observed more in males (51.4%); normal (41%) and short (39.3%) facial morphology were more common in females. The measurements and proportions for facial balance in our study population showed that the facial width and height proportions deviated from the golden proportion.

## Resumo

**Introdução:**

A face é o aspecto mais importante da aparência física de uma pessoa. Na estética facial, existe uma proporção matemática específica, chamada de proporção áurea. A proporção áurea é usada para medir e analisar as qualidades estéticas da face na população.

**Objetivo:**

Medir as proporções dos tecidos moles faciais que contribuem para o padrão da beleza facial, auxiliar a percepção e diagnóstico das diferenças e anomalias faciais e comparar essas proporções com a proporção áurea.

**Método:**

Foram incluídos no estudo 133 pacientes turcos com 18 a 40 anos (61 mulheres, 72 homens). A análise das fotografias foi realizada pelo mesmo médico e um programa de *software* foi usado (NIH Image, versão 1.62). As proporções faciais foram medidas e as diferenças das proporções áureas foram registradas e agrupadas como normais (1,6–1,699), curtas (<1,6) e longas (>1,699).

**Resultados:**

De acordo com os resultados da análise facial, avaliou-se a proporção do tríquion-gnátio/zigoma direito-zigoma esquerdo e 33,1% dos pacientes apresentaram morfologia facial normal, enquanto 36,8% tinham morfologia facial longa e 30,1% morfologia facial curta, segundo essa proporção. A proporção do tríquion-gnátio/zigoma direito-zigoma esquerdo, foi significantemente maior em homens do que em mulheres (p < 0,001). Uma diferença estatisticamente significante foi observada entre os sexos, de acordo com a proporção do tríquion-gnátio/zigoma direito-zigoma esquerdo e do canto lateral direito-canto lateral esquerdo/ângulo cantal direito- ângulo cantal esquerdo (p = 0,001, p = 0,028).

**Conclusão:**

A avaliação da proporção facial em relação à proporção áurea mostrou que houve diferença estatisticamente significante entre os sexos. A morfologia facial longa foi mais observada no sexo masculino (51,4%), a morfologia facial normal (41%) e a curta (39,3%) foram mais comuns no sexo feminino. As medidas e proporções para o equilíbrio facial em nossa população estudada mostraram que as proporções de largura e altura faciais se desviaram da proporção áurea.

## Introduction

The face is the most important factor affecting the physical appearance of a person.^1^ The most important factors of facial attractiveness are averageness, sexual dimorphism, youthfulness and symmetry.^2^ In addition, the role of smile in general facial aesthetics has been investigated in the literature.^3^, ^4^

**Figure 1:**
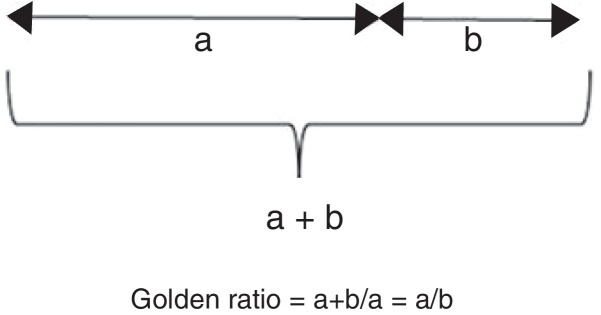
Calculation of GP. GP divides a line into two parts (a, b), and proportion of the two parts (a/b) is equal to the proportion of the total length to the longer part (a + b/a). G.P.’s value is equal to 1.618.

In facial aesthetics, there is a specific mathematical proportion which is called golden proportion (GP).^5^ The GP is a commonly observed identity in nature. In the fourth-century A.C., Euclid described the GP geometrically, which divides a line into two parts (a, b), and the proportion of the two parts (a/b) is equal to the proportion of the total length of the longer part (a + b/a) (Fig. 1). Geometrically described by Euclid, and also called the Fibonacci proportion, or “Divine Proportion”, GP's value is equal to irrational number called “phi” (1.618), which is named after Parthenon Phidias.^6^, ^7^ Clinical applications of the GP are mostly employed in dental prostheses, aesthetic surgery, orthodontics and facial mask fields. The GP is used to evaluate the aesthetic appearance of face in aesthetic surgery.^8^ Many authors have used the GP tool to measure and analyse facial aesthetic qualities in their own countries.

The aim of the present study was to measure the facial soft tissue proportions which would help to realize and diagnose facial differences and anomalies and to compare these proportions to the GP in our population.

## Methods

This study was conducted from January 2016 to January 2017 in Sisli Hamidiye Etfal Training and Research Hospital, Istanbul. Ethics committee's approval number is 1186.

One hundred and thirty-three (133) Turkish patients 18–40 years of age (61 females, 72 males) were involved in the study. Patients who had any previous facial trauma and facial surgery were excluded from the study.

Every face changes as it grows, and there are many variations in view of this change. Therefore, we included patients between the ages of 18 and 40. Male patients were asked to shave before photographing, to make sure that beard or moustache did not affect the measurements.

**Figure 2:**
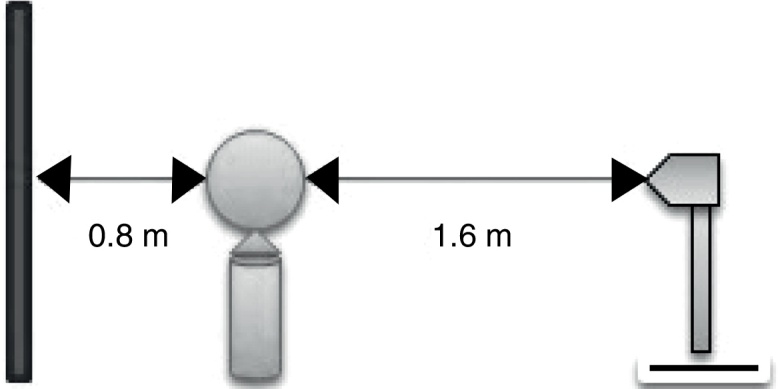
Distances of taking a photo. Photographs were taken from a constant standpoint from the patient.

Routine otolaryngology examination was performed on every patient. Photographs were taken in the studio of our hospital by a professional photographer from a constant distance point from the patient, and Canon EOS 500D (1/60, f/5 70 mm) was used (Fig. 2).

**Figure 3:**
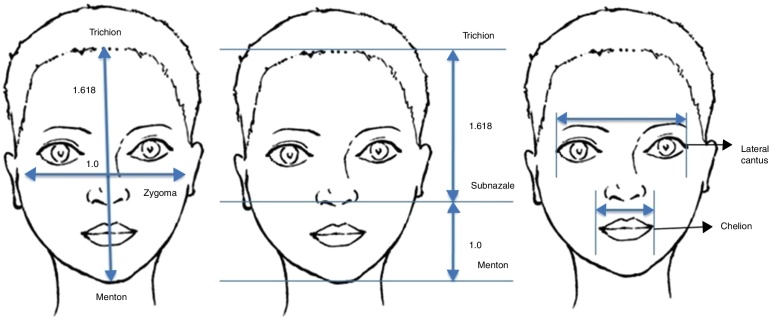
Measurements and ratios. Anatomical points, measurements and proportions which are used for facial analysis.

Photographs were in a standard size (5″ × 4″), and the analysis was performed by a single physician. A software programme was used for measuring. Anatomical points, measurements and proportions which are used for the analysis are detailed in Table 1 and Fig. 3.

**Table 1:** Anatomical points, measurements and proportions.

Points	Face height measurements	Face width measurements	Proportions
Trichion (Tr)	Tr–Gn	Zg–Zg	Tr–Sn/Sn–Gn
Lateral canthus (Lc)	Tr–Sn	LcR (right)–LcL (left)	Tr–Gn/Zg–Zg
Zigoma (Zg)	Sn–Gn	ChR (right)–ChL (left)	LcR–LcL/ChR–ChL
Subnasal (Sn)			
Cheilion (Ch)			
Gnathion (Gn)			

Trichion (Tr), junction point of the upper part of the forehead (hairline); Lateral canthus (Lc), point in lateral canthus of eyes; Zigoma (Zg), lateral point of the zygomatic arc; Subnasal (Sn), the intersection point of the upper lip and nasal septum; Cheilion (Ch), point in corner of the mouth; Gnathion (Gn), the lowest point in the middle of the soft tissue of the mentum.

In this study, physiognomic facial height and width were measured, and the mean value in both genders was recorded. The percentage difference from the GP was calculated by “percentage difference = |Δ*V*|/(Σ*V*/2) × 100″ formula. Results were evaluated and patients were grouped according to facial morphologies in vertical and horizontal craniofacial planes (1.6–1.699 = normal, <1.6 = short, >1.699 = long).

### Statistical analysis

SPSS software was used for statistical analysis. Definitive statistics for numerical variables were given as mean, standard deviation and 95% CI minimum and maximum, for categorical variables, were given as numbers and percentage. In two independent groups, numerical variables were analysed by Student's *t*-test. Chi-square was used for the comparison of ratios. Monte Carlo simulation was used when requirements were not provided. Statistical alpha level was accepted as *p* < 0.05.

## Results

Facial height (Tr–Sn/Sn–Gn) and facial wideness (LcR–LcL/ChR–ChL) measurements showed that patients predominantly showed long face morphology (Table 2).

**Table 2:** Mean values of facial analysis ratios.

	Mean ± SD	95% CI
Tr–Gn/ZgR–ZgL	1.65 ± 0.10	1.64–1.67
Tr–Sn/Sn–Gn	1.83 ± 0.18	1.80–1 .86
LcR–LcL/ChR–ChL	1.88 ± 0.13	1.86–1.90

According to the Tr–Gn/ZgR–ZgL facial analysis proportions, facial morphologies were 33.1% normal, 36.8% long and 30.1% short. The Tr–Sn/Sn–Gn facial analysis proportions showed that 13.5% of the patients were in normal morphology, 75.2% were in long morphology and 11.3% were in short morphology. In reference to the LcR–LcL/ChR–ChL, facial analysis revealed that 6.8% of the patients were in normal morphology, 92.5% were in long morphology, while 0.8% were in short morphology (Table 3). The Tr–Gn/ZgR–ZgL proportions were significantly higher in males and the Tr–Sn/Sn–Gn proportions were significantly lower (*p* < 0.001, *p* = 0.009). The Tr–Gn/ZgR–ZgL proportions were significantly higher in males than in females, the Tr–Sn/Sn–Gn proportions were significantly lower in males (*p* < 0.001, *p* = 0.009). No significant difference was noted between the gender groups in the LcR–LcL/ChR–ChL proportions analysis (*p* = 0.075) (Table 4).

**Table 3:** Face morphology results according to facial analysis rates.

	Tr–Gn/ZgR–ZgL		Tr–Sn/Sn–Gn		LcR–LcL/ChR–ChL	
	*n*	%	*n*	%	*n*	%
Normal (1.600–1.699)	44	33.1	18	13.5	9	6.8
Long (>1.699)	49	36.8	100	75.2	123	92.5
Short (<1.6)	40	30.1	15	11.3	1	0.8

**Table 4:** Mean values of face analysis ratios by gender.

	Male		Female		*p*
	Mean ± SD	95% CI	Mean ± SD	95% CI	
Tr–Gn/ZgR–ZgL	1.69 ± 0.10	1.67–1.71	1.61 ± 0.09	1.59–1.64	<0.001
Tr–Sn/Sn–Gn	1.79 ± 0.17	1.75–1.83	1.87 ± 0.18	1.83–1.92	0.009
LcR–LcL/ChR–ChL	1.86 ± 0.13	1.83–1.89	1.90 ± 0.13	1.87–1.94	0.075

A statistically significant difference was noted in gender groups, according to the Tr–Gn/ZgR–ZgL and the LcR–LcL/ChR–ChL facial analysis proportions (*p* = 0.001, *p* = 0.028). In the Tr–Gn/ZgR–ZgL facial analysis proportions, males tended to have longer facial morphology, whereas in the LcR–LcL/ChR–ChL proportions, females tended to have longer facial morphology (Table 5).

**Table 5:** Face morphology results by gender facial analysis rates.

		Male		Female		*p*
		*n*	%	*n*	%	
Tr–Gn/ZgR–ZgL	Normal (1.600–1.699)	19	26.4	25	41.0	0.001
	Long (>1.699)	37	51.4	12	19.7	
	Short (<1.6)	16	22.2	24	39.3	
Tr–Sn/Sn–Gn	Normal (1.600–1.699)	1	15.3	7	11.5	0.195
	Long (>1.699)	50	69.4	50	82.0	
	Short (<1.6)	11	15.3	4	6.6	
LcR–LcL/ChR–ChL	Normal (1.600–1.699)	8	11.1	1	1.6	0.028
	Long (>1.699)	64	88.9	59	96.7	
	Short (<1.6)	0	0.0	1	1.6	

Facial analysis proportions’ percentage difference from the GP (1.618) was depicted in Table 6.

**Table 6:** Facial analysis proportion's percentage difference from the GP.

	Golden ratio percentage of differences	
	Mean ± SD (%)	95% CI
Tr–Gn/ZgR–ZgL	2.0 ± 6.4	0.9–3.0
Tr–Sn/Sn–Gn	11.8 ± 9.9	10.1–13.5
LcR–LcL/ChR–ChL	14.8 ± 6.9	13.6–16.0

The Tr–Gn/ZgR–ZgL facial analysis proportion's percentage difference from the GP was significantly higher in males than in females, Tr–Sn/Sn–Gn proportion's difference from the GP was significantly lower in males (*p* < 0.001, *p* = 0.010). LcR–LcL/ChR–ChL facial analysis proportion's percentage difference from the GP was not significantly different between the gender groups (*p* = 0.075) (Table 7).

**Table 7:** Percentage difference of facial analysis ratios to golden ratio by gender.

	Male		Female		
	Mean ± SD	95% CI	Mean ± SD	95% CI	*p*
Tr–Gn/ZgR–ZgL	4.1 ± 6.0	2.7–5.5	−0.6 ± 6.0	−2.1 to 1.0	<0.001
Tr–Sn/Sn–Gn	9.8 ± 9.7	7.5–12.1	14.2 ± 9.6	11.8–16.7	0.010
LcR–LcL/ChR–ChL	13.8 ± 7.0	12.1–15.4	16.0 ± 6.6	14.3–17.6	0.075

## Discussion

The concept of aesthetic has changed over time, thus it is hard to define beauty and ideal aesthetic features. Attractiveness is a subjective entity which is influenced by age, race, gender, ethnicity and educational level of a person.^9^

The definition of beauty has been explored since ancient Egyptian civilization. Euclid, Pythagoras, Vitruvius and Leonardo Da Vinci tried to define the beauty with mathematical algorithms. These algorithms played a significant role in the definition of beauty and facial attractiveness. The GP is a well-known example of these algorithms.^6^, ^7^, ^9^

There are many studies in the literature about the association of GP and aesthetic perception. Segher et al. first described the use of the GP in facial aesthetic surgery.^10^ Rickets is the first orthodontist who used the GP for composition of soft and hard tissues of the face.^8^ Marquardt used the GP to develop a facial mask to define the structural balance of the face.^11^

The application of the GP has showed different results in several studies. Kawakami et al.,^12^ Filho et al.,^13^ Mizumoto et al.^8^ and Sunilkumar et al.^14^ reported that there exists a relationship between divine proportion and facial aesthetics. Kiekens et al.^7^ reported that proportions of attractive faces are closer to the GP. However, Rossetti et al.^15^ showed that there was no correlation between the perception of facial beauty and the divine proportion.

**Figure 4:**
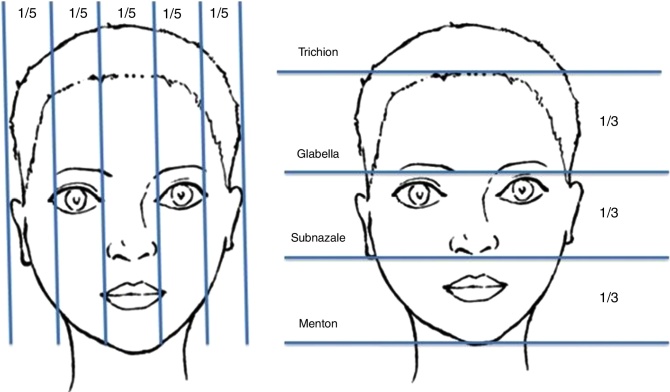
Height and width ratios of a face. A face is divided into five equal parts in the vertical plane and three equal parts in the horizontal plane.

The face is divided into three parts in the horizontal plane. The upper part is in between the trichion and glabella, the middle part is in between the glabella and subnasal, and the lower part is in between the subnasal and mentum (Fig. 4). These three parts should be equal ideally, but commonly these parts are not equal. Studies about facial height proportions reported that there is only 50% of equality.^1^

Facial height is higher in males than in females in all races. However, Farkas et al.^16^ conducted a study in the Indian population, which demonstrated that females had higher facial heights. In a study by Packiriswamy et al.,^10^ in 229 of 300 Malaysian people, short facial morphology was detected.

In our study, facial analysis results showed that long facial morphology was significantly higher (Table 3). According to the Tr–Sn/Sn–Gn facial analysis, proportion's percentage difference from the GP was significantly lower in males than in females (*p* = 0.010, 95% CI 7.5–12.1) (Table 7).

The face is divided into five parts in vertical plane. The ideal width of every eye is one part, each of two intercanthal distances and nasal wideness comprises one part each (Fig. 4). Width of the lips should be 40% of the lower face and should be equal to the distance between the medial limbi. Previous studies reported that in Caucasian and Asian population, there are differences in these proportions: width of the eyes and nose were found to be greater or narrower than intercanthal distance.^1^

According to facial analysis results, wide facial morphology was observed more than long facial morphology (Table 3). According to the LcR–LcL/ChR–ChL, percentage difference from the GP was not significantly different in gender groups (Table 7).

Farkas et al. reported that American, Afro-American, Caucasian, Malaysian, Indian, Arabic and Chinese people have different facial characteristics, which is affected by race and ethnicity. In Table 8, facial characteristics including facial height and facial wideness in different races are summarized.^16^, ^21^ Farkas et al. also reported that males had longer facial morphology than females in the Turkish population. In our study, according to the Tr–Gn/ZgR–ZgL, males had longer facial morphology which was consistent with the previously reported data (Table 3). According to the Tr–Gn/ZgR–ZgL proportions, percentage difference from the GP was significantly greater in males than in females (*p* < 0.001, 95% CI 2.7–5.5) (Table 7).

**Table 8:** Comparison of face measurements at different populations in the world.

Author	Year	M	F	Population	Method	Height of face		Width of face	
						M	F	M	F
Farkas et al.^16^	2005	30	30	North American	Caliper	187.5 ± 16.2	172.5 ± 15.0	137.1 ± 8.6	129.9 ± 10.6
		30	30	Azerbaijan		185.1 ± 18.0	175.4 ± 13.6	147.5 ± 10.8	138.7 ± 10.4
		30	30	Bulgarian		184.3 ± 17.4	170.5 ± 13.6	139.5 ± 11.2	130.9 ± 8.8
		30	30	Czech		181.7 ± 15.8	182.9 ± 16.2	134.9 ± 26.6	126.4 ± 28.8
		30	30	Croatian		180.1 ± 21.2	172.6 ± 17.4	140.7 ± 12.0	133.2 ± 13.6
		30	30	German		182.2 ± 22.2	170.9 ± 14.4	133.2 ± 15.0	123.4 ± 18.4
		30	30	Greek		178.7 ± 25.8	173.8 ± 13.8	128.6 ± 22.8	132.2 ± 9.6
		30	30	Hungarian		181.3 ± 28.4	169.4 ± 15.4	142.1 ± 10.6	131.3 ± 7.0
		30	30	Italian		186.0 ± 21.2	171.4 ± 18.4	143.2 ± 11.8	133.3 ± 8.2
		30	30	Polish		181.9 ± 16.4	172.1 ± 17.8	142.6 ± 9.4	135.5 ± 11.0
		30	30	Portuguese		190.7 ± 14.2	177.4 ± 19.0	125.1 ± 10.8	120.4 ± 10.8
		30	30	Russian		184.4 ± 16.2	174.4 ± 17.4	141.2 ± 8.8	132.3 ± 9.6
		30	30	Slovak		183.7 ± 17.6	169.7 ± 17.5	134.7 ± 11.0	125.0 ± 11.4
		30	30	Slovenian		181.3 ± 20.6	170.4 ± 30.2	136.2 ± 11.6	129.5 ± 10.4
		30	30	Iranian		180.3 ± 20.4	175.9 ± 15.0	138.4 ± 11.4	131.7 ± 13.4
		30	30	Turkish		186.5 ± 12.8	179.2 ± 18.8	140.4 ± 16.4	134.5 ± 8.6
		30	30	Egyptian		176.9 ± 26.8	161.4 ± 17.8	139.8 ± 13.8	130.3 ± 10.4
		30	30	Indian		161.3 ± 4.6	163.0 ± 16.6	135.8 ± 8.6	124.9 ± 16.9
		30	30	Singapore Chinese		187.3 ± 14.4	176.2 ± 16.6	144.6 ± 11.2	136.2 ± 8.0
		30	30	Vietnamese		180.9 ± 16.6	171.1 ± 14.2	144.0 ± 8.8	134.3 ± 5.8
		30	30	Thai		185.1 ± 15.4	172.8 ± 17.4	147.1 ± 11.0	138.3 ± 12.6
		30	30	Japanese		191.4 ± 16.6	182.8 ± 14.4	147.2 ± 11.2	141.2 ± 11.8
		30	30	Angolan		182.6 ± 18.2	172.4 ± 17.8	139.8 ± 10.2	132.8 ± 8.4
		30	30	Tonga		161.8 ± 17.0	—	133.3 ± 2.6	—
		30	30	Zulu		209.2 ± 20.6	179.1 ± 19.8	138.5 ± 9.2	128.4 ± 9.6
		30	30	Afro American		194.6 ± 21.2	180.1 ± 15.0	138.7 ± 11.2	130.5 ± 9.6
Erika et al.^16^	2005	39	38	Latvian	Caliper	187.3	177.0	133.1	122.4
Omar et al.^17^	2005	—	102	Indian American	Photograph	—	169.4 ± 13.3	—	125.9 ± 10.1
Ngeow et al.^18^	2009	50	50	Malay	Caliper	—	—	132.5 ± 7.0	140.1 ± 4.9
Ngeow et al.^19^	2009	50	50	Malaysian Indian	Caliper	—	—	136.3 ± 4.8	126.7 ± 3.9
Raji et al.^16^	2010	200	143	North Eastern Nigerian	Caliper	—	—	115.1	111.3
Jeremic et al.^16^	2013	360	340	Serbian	Caliper	—	—	129.1 ± 8.9	120.0 ± 6.4
Kumar et al.^16^	2013	300	300	Haryanvi Bania	Caliper	—	—	130.8 ± 7.3	123.5 ± 7.6
Milutinovic et al.^20^	2014	—	83	Caucasian	Photograph	—	—	141.7 ± 18.8	—
Packiriswamy et al.^10^	2012	50	50	Malaysian Chinese	Caliper	192.1 ± 9.6	186.6 ± 9.9	140.1 ± 7.4	135.2 ± 10.8
		50	50	Malaysian Indian		182.5 ± 11.0	172.7 ± 10.9	130.3 ± 8.9	124.0 ± 6.6
		50	50	Malay		189.1 ± 8.4	179.2 ± 7.8	131.3 ± 8.7	134.0 ± 10.2
Alam et al.^21^	2014	50	50	Malaysian Chinese	Caliper	188.4 ± 14.0	172.6 ± 22.5	117.1 ± 11.5	115.2 ± 13.4
		36	50	Malaysian Indian		178.3 ± 13.2	168.3 ± 13.5	112.7 ± 9.6	107.8 ± 13.8
		50	50	Malay		179.1 ± 15.3	161.8		114.8 ± 10.1

Packiriswamy et al. conducted a study in 300 people and reported that Tr–Gn/ZgR–ZgL facial analysis proportion's percentage difference from the GP showed that 229 people had short facial morphology and 23 people had long facial morphology.^10^ In our study, males had long facial morphology (51.4%), whereas females had normal (41%) and short (39.3%) facial morphology (Table 5).

In the literature, many authors reported that beauty is affected by many factors, such as genetic, cultural and environmental factors. Facial beauty can be assessed literally by global parameters such as neoclassic laws and the GP, and faces with different characteristics can be found attractive in different cultures and ethnic groups. However, these proportions are not the only factors affecting facial attractiveness.

## Conclusion

In our study, facial morphologies were significantly different in gender groups. Facial morphologies of male and female populations were found to be predominantly shorter and longer: 73.6% in males, short face (22.2%) and long face (51.4%) and, 59% in females, short face (39.3%) and long face (19.7%).

The measurements and proportions for facial aesthetic in our study population showed that the facial width and height proportions deviated from the GP. Further studies are needed in order to evaluate the general population.

## Conflicts of interest

The authors declare no conflicts of interest.
